# Synthesis of a new biocomposite for fertiliser coating: assessment of *biodegradability* and *thermal stability*

**DOI:** 10.1007/s11356-023-28892-0

**Published:** 2023-07-29

**Authors:** Mohammad Reza Ketabchi, Salman Masoudi Soltani, Andy Chan

**Affiliations:** 1 Department of Chemical and Environmental Engineering, Faculty of Engineering, University of Nottingham Malaysia, 43500 Semenyih, Selangor Malaysia; 2 Department of Chemical Engineering, Brunel University London, Uxbridge, UB8 3PH UK; 3 School of Engineering, Robert Gordon University, Aberdeen, AB10 7GJ UK

**Keywords:** Biocomposites, Biodegradation, Water absorption, Thermal stability, Kinetics

## Abstract

The bio- and thermal degradation as well as the water absorption properties of a novel biocomposite comprising cellulose nanoparticles, natural rubber and polylactic acid have been investigated. The biodegradation process was studied through an assembled condition based on the soil collected from the central Malaysian palm oil forests located in the University of Nottingham Malaysia. The effects of the presence of the cellulose nanoparticles and natural rubber on the biodegradation of polylactic acid were investigated. The biodegradation process was studied via thermal gravimetric analysis and scanning electron microscopy. It was understood that the reinforcement of polylactic acid with cellulose nanoparticles and natural rubber increases the thermal stability by ~ 20 °C. Limited amorphous regions on the surface of the cellulose nanoparticles accelerated the biodegradation and water absorption processes. Based on the obtained results, it is predicted that complete biodegradation of the synthesised biocomposites can take place in 3062 h, highlighting promising agricultural applications for this biocomposite.

## Introduction

Biodegradable materials are increasingly becoming popular—owing to their inherently safer disposal pathways. Many industries including packaging, biomedical, pharmaceutical, agricultural and horticultural, textile, household goods and automotive have been integrating the use of biodegradable materials into their end products (Nanda et al. 2021; Imbachi-Hoyos et al. 2022). According to the USA Biodegradable Products Institute (BPI) standards, biomaterials are classed as those which must disintegrate within 3-month (2160 h) of their production (Briassoulis et al. 2010).

Degradation involves fragmentation and changes in the molecular arrangements of materials. There are different types of degradation, e.g. thermal degradation, thermos-oxidative degradation, direct and indirect photo-degradation, irradiation degradation, mechano-chemical degradation, chemical degradation and biodegradation (Teixeira et al. 2021; Rohidi and Othman 2022; Pires et al. 2022). The degradation of biodegradable materials disposes various organic resources which could benefit the soil. During the natural degradation process (biodegradation), the process begins with photodegradation by UV light from the sun and the organic constituents are oxidised and converted to ecologically accepted molecules including minerals, water and carbon dioxide (Chellasamy et al. 2022). According to the EN-13432 European standard, in the absence of oxygen, these biomaterials decompose into minerals, carbon dioxide and methane (Notaro et al. 2022). These soil-friendly features have gained considerable attention among agricultural sectors. Biocomposites have both directly and indirectly (as a carrier) shown their applicability in the improvement of soil properties in several distinct ways (Akhir and Mustapha 2022).

Farmers have been using fertilisers for many decades to improve soil properties for enhanced cultivation. Fertilisers contain hydrophilic minerals and salts. Fast dissolution of these salts causes irrecoverable damages to both soil and plants’ nutrients’ uptake. To control the dissolution process, various methods have been investigated. One method is introduction of compounds with low water solubility and low volatility to slow down the dissolution process (Barra Caracciolo and Grenni 2022). Through this method, the synthesised compounds release the nutrients over longer periods of time (slow release). This can be costly in practice, depending on the soil condition (temperature, oxygen level, pH) and plant’s needs (Malathi et al. 2021). An alternative method to overcome this is to encapsulate the actual fertiliser with organic compounds capable of progressively releasing the nutrients following its biodegradation in soil (Angelo et al. 2021; Javazmi et al. 2021). This results in gradual release of the nutrients synced with the continual biodegradation process. Depending on the target application, the biodegradation can be further tuned by adjusting the capsule thickness or employing more/less rigid compositions. This, in return, builds on more confidence in commercial viability of such biocomposites (Tripathi et al. 2021).

Biopolymers are extracted from natural resources such as corn, potato and sugarcane (Corrêa 2022). They have become an alternative to petroleum-based plastics following their global availability and the facile cultivation of natural resources. Through different agricultural environments, plants are cultivated, harvested and then chemically and physically treated to extract their starch content. The isolated starch is then refined using special enzymes to produce a specific biopolymer (Lasprilla et al. 2012; Apriyanto et al. 2022). This product is fully biodegradable into mainly water and carbon dioxide. Biodegradable polymers are either plant- and bacterial-based or synthesised using a bio-based monomer. Polylactic acid (PLA) is a synthesised biopolymer comprising lactic acid monomers (Ahmad et al. 2022). However, poor thermal and mechanical performance and also high production costs are the key drawbacks in large-scale applications of the PLA (Taib et al. 2022).

PLA has been reported to be well biodegradable; however, it suffers from low degradation rates when compared to other aliphatic biopolymers (Rosli et al. 2021). In the early stages of PLA degradation, the PLA chains with high molecular weight (ester bonds) are hydrolysed to form lower molecular weight chains (Fukushima et al. 2012). Amorphous regions provide higher hydrolysis rate compared to the crystalline counterparts (Sun et al. 2013). An increase in the biodegradation rate was observed following reinforcement of PLA with starch (an inherently amorphous material) which was ascribed to moisture absorption which benefits the hydrolysis process (Wu 2005, Hernández-Carmona et al. 2017). On the other hand, an increase in the PLA crystallinity was reported to decrease its biodegradation rate (Urayama et al. 2002; Shogren et al. 2003). The introduction of a basic or an acidic solution to the soil and an elevation in both the temperature and the humidity were found to facilitate these reactions (Avinc et al. 2010). Degradation and mineralisation of the PLA has been stated to complete in 6480 h (9 months) and 84 h at 30 °C and 70 °C, respectively (Lunt 1998). The biodegradation rate of the PLA plastic films in soil was reported at 28 mg in 24 h at 28 °C with 27% conversation to CO_2_ (Ho and Pometto 1999). Higher degradation rates were observed at the sample core due to the high concentration of carboxylic acid groups which enhance the ester hydrolysis (Harmaen et al. 2015). Meanwhile, bacterial and fungal microbes can act as a catalyser (Wilkes and Aristilde 2017). Such microorganisms (polysaccharide utilisers) break ester bonds to an acid or an alcohol, which then form low-molecular-weight chains which, in the end, convert into CO_2_, water and humus (Seshikala and Charya 2012). Development of fungal mycelia on the PLA surface was reported after 8 weeks in soil (Shogren et al. 2003).

In this study, a new biocomposite was prepared as an encapsulating material for fertilisers. The biodegradation and water absorption behaviour of the synthesised biocomposite was determined (ASTM D256) and a polynomial model was developed.

## Experimental procedures

### Materials

Polylactic acid (Ingeo biopolymer) (grade 2003 D; melt index: 6 g/10 min; density: 1.22 g/cm^3^) was supplied by Nature Works LLC product, USA. The granules were dried at 60 °C for 12 h prior to the compounding process. Synthetic liquid polyisoprene (LIR-30) (Molecular weight: 28,000, Tg: − 63 °C by Kuraray Co. Ltd., Japan) was used as the natural rubber (NR). Cellulose nanoparticles (CNPs) were extracted from kenaf fibre according to the optimised process obtained through our earlier study (Ketabchi et al. 2016). The soil in this study was collected from the central Malaysian palm oil forests based in the University of Nottingham Malaysia. The soil had a pH of 6.5 (1:2 soil and water suspension) and did not contain any composting materials and showed no enzymatic activity.

### Methods

#### Compounding process

Nine different sets of compositions were prepared via melt compounding, followed by injection moulding (Table 1). All biocomposites were prepared using a Brabender PL2000-6 twin-screw compounder (180 °C, 10 min and 100 rpm). All compounds were then moulded using a bench-top injection moulding machine (RR3400, RAY-RAN Injection Moulding Machine). The barrel temperature was set at 180 °C, and the mould temperature was set at 90 °C. Holding time was set at 8 s in accordance with the ASTM D256 method.

**Table 1:** The compositions of the prepared samples

Sample	PLA content (wt%)	CNP content (wt%)	NR content (wt%)
PLA	100	–	–
PN1	99	1	–
PN2	97	3	–
PN3	95	5	–
PR1	95	–	5
PR2	90	–	10
PR3	85	–	15
PR4	80	–	20
PNR	87	3	10

#### Biodegradation test

The biodegradation process of the PLA-based biocomposites was carried out in a natural-soil environment, using accessible tools and materials (Harmaen et al. 2015). The soil was free from any enzymatic activities and any compositing additive. Nine plastic containers (250 ml) were filled with identical amounts of soil samples (labelled according to Table 1). The biocomposite samples (~ 30 × 10 × 3 mm) were then placed (buried) into each of the nine containers. The containers were next kept at room temperature (28 °C) and with an ambient relative humidity of 80%. The experiment was continuously run for 2160 h. The biocomposites were subsequently weighed using a lab-scale balance. The change in the biocomposites’ masses was determined after 6, 12, 24, 48, 72, 96, 120, 1440, 1680, 1920 and 2160 h after their initial burial in soil samples. Prior to any analysis, each biocomposite was first rinsed under tab water to wash out the soil residues and later dried at 80 °C to fully remove the moisture until a constant dry weight was achieved. The initial (i.e. before test) and the secondary (i.e. after x hours) sample weights were recorded and labelled as *S*_0_ and *S*_1_, respectively. The weight loss percentage was calculated using Eq. 1:

1 $$\mathrm{Weight\;loss }(\mathrm{\%})=\frac{S0 - S1}{S0 }\times 100$$

#### Water absorption test

The test was done according to the ASTM D 570 (Hosseinihashemi et al. 2016). Plastic containers were used and filled with distilled water. The containers were kept at room condition (28 °C, 80% humidity). The water level was maintained the same throughout the entire test run. The biocomposites were weighed initially and after 6, 12, 24, 48, 72, 96, 120, 1440, 1680, 1920 and 2160 h of soaking in distilled water. Prior to each analysis, biocomposites were moderately dried using a towel to remove the excess water on the surface until a constant weight was obtained. The primary (before test) and secondary (after x hours) weights of the samples were recorded and labelled as *W*_0_ and *W*_1_, respectively. The water absorption percentage was calculated using Eq. 2:

2 $$\mathrm{Water\;absorption\;}(\mathrm{\%})=\frac{W0 - W1}{W0}\times 100$$

#### Thermogravimetric analyses

Both of the non-biodegraded and biodegraded samples were used to determine the influence of biodegradation on the thermal stability of the biocomposites. Perkin Elmer simultaneous thermal analyser (STA 6000, USA) was used to run the themo-gravimetric analyses (nitrogen flow rate: 10 ml/min; temperature starting from 30 to 500 °C, a heating rate of 10 °C/min).

#### Scanning electron microscopy

Biocomposites were recovered from soil after 2160 h of burial and their surface structure was analysed using a scanning electron microscope (FEI QUANTA 400F) at 20 kV. The surface morphologies were studied and compared with the un-buried biocomposite samples.

## Results and discussion

### Biodegradation in soil

**Fig. 1:**
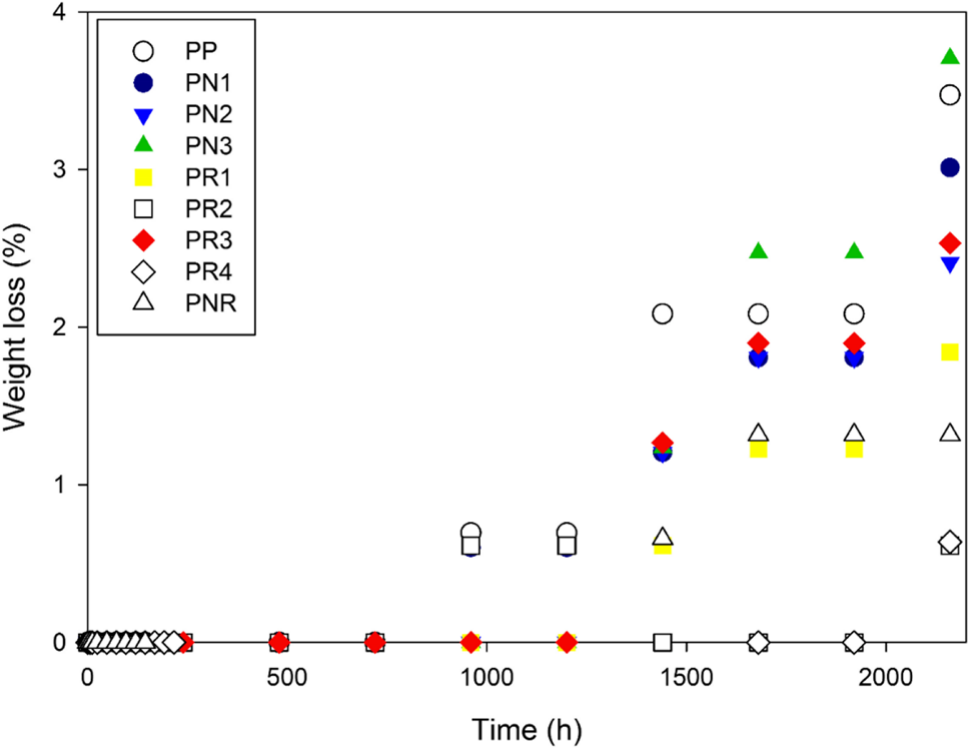
Weight loss% of the biocomposites in the soil test with respect to time

The results show that the biocomposites lose their weight by 0.61 to 3.70% (Fig. 1). Within the first 720 h of the burial in soil, the biocomposites were only slightly decomposed. Within the same test period, the weight of the PN2, PN3, PR1, PR4 and PNR increased by only 0.6%. This is associated with water uptake from the soil. A weight loss of just 0.61–2.08% was observed at the very early stage of the degradation process (i.e. after 1440 h). The biodegradation process was more noticeable after 2160 h of burial where CNP/PLA biocomposites underwent a weight loss of ~ 3%. However, the NR/PLA biocomposites lost their weight by about 1.5%. Basically, a higher CNP and a lower NR content resulted in a faster biodegradation process. Consequently, the PN3 corresponded to the fastest observed biodegradation process. This is linked to the amorphous regions present in CNP which favourably affects the biocomposites’ resistance against degradation. CNP contains both crystalline and amorphous regions; however, a dominant crystalline region was detected as a result of the experimental conditions. Fair biodegradation of the biocomposites with CNP confirms the fact that amorphous structures are more easily attacked by hydrolytic enzymes which can result in much higher biodegradation rates (Liu et al. 2022). This suggests the significant role of the amorphous regions in the biodegradation processes. Depending on the applications, further CNP treatment can be considered in order to tailor-make the degree of crystallinity.

The decomposition of the PR4 was slower than that of any other studied biocomposites. This is associated with the hydrophobic nature of the NR which retains the PLA structure. A similar trend was also observed with the PNR which was linked not only to the nature of the NR but also to the enhanced interfacial adhesion between the PLA matrix and the CNP due to the addition of the NR. Therefore, the PNR composition exhibited a moderate degradation phase as compared to both the CNP/PLA and the NR/PLA biocomposites. The PNR showed a weight loss of 1.3% after 2160 h of the initial burial.

### Water absorption

**Fig. 2:**
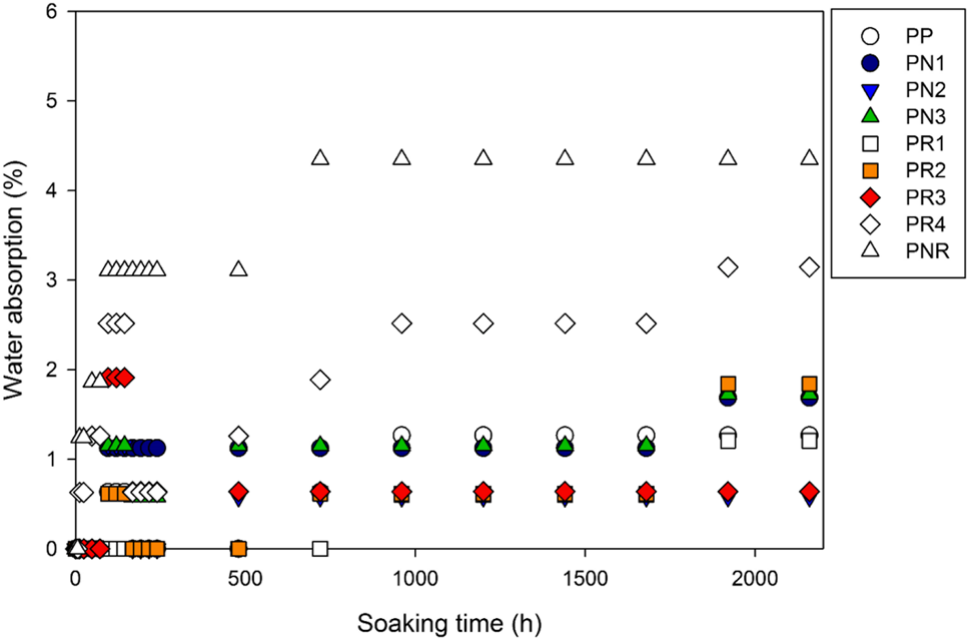
Water uptake as a function of exposure time in distilled water

Water absorption test was carried out in parallel with the soil burial test (Fig. 2). During the first 72 h of the test, most of the samples appeared to absorb only little amount of water. However, the PR4 and PNR samples began a considerable water absorption after 12 h. They both demonstrated the highest water uptake during the test: 3.14% for PR4 and 4.34% for PNR after 2160 h. The biocomposites with CNP had relatively lower water absorption rates as compared to the biocomposites with NR. This is linked to the strong crystalline structure of CNP and the preparation technique, minimising the hydrophilic nature of the fibre. Therefore, an increase in the CNP content shows a minor impact on the water absorption rate. On the other hand, an increase in the NR content, elevates water uptake. This is linked to the excess amount of NR in the PR4 which results in the formation of micro bubbles and voids in the sample. The performance of composite materials is highly dependent on the presence of pores as they influence the heat and mass transfer behaviour (Hosseinihashemi et al. 2016). Most of the biocomposites showed a maximum water absorption of about 1% after 2160 h (expect for the PR4 and PNR). The PN2 and PR3 were associated with the lowest water absorption rates. This reveals that a combination of CNP (3 wt%) and NR (15%) is the optimum amount of reinforcement in order to minimise the PLA’s water absorption.

### Thermal degradation

**Fig. 3:**
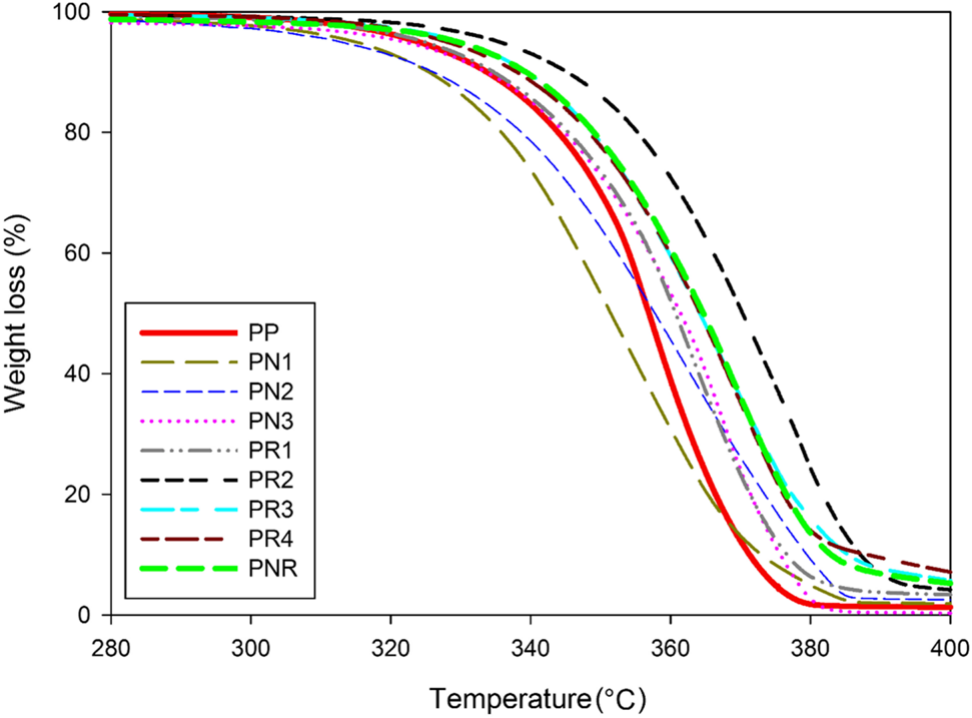
Thermogravimetric analyses: PLA and the biocomposites

The thermal stability of the biocomposites was analysed via thermogravimetric analyses (TGA) analyses. Figure 3 illustrates the weight loss during the TGA test. All biocomposites began to degrade at about 320 °C. At this temperature, the PN1 and PN2 both showed an 8% weight loss while the PN3 underwent a 5% weight loss. This trend became more noticeable at temperatures > 360 °C. At this temperature, the PN1 showed a 70% weight loss compared to a 55% and a 48% weight loss for the PN2 and PN3, respectively. This reveals the effect of CNP on the thermal stability of the PLA at high temperatures. Meanwhile, the biocomposites with NR showed an opposite behaviour compared to the biocomposites with CNP.

Higher concentrations of NR (> 10 wt%) adversely impacts the thermal stability of the PLA. The excess NR was located between the PLA granules, i.e. creating a gap between the granules, which in turn affects the overall stability of the PR3 and PR4 at higher temperatures. Compared to NR, at such elevated temperatures, weight loss is mainly associated with the degradation of the cellulose fibres. Therefore, the PR2 shows the highest thermal stability among the biocomposites. The thermal studies together with the soil burial and water absorption tests successfully pinpointed the optimum PNR compositions (i.e. 3 wt% CNP and 10 wt% NR). The reinforcement of PLA with both NR and CNP improved the thermal stability by almost 20 °C.

**Fig. 4:**
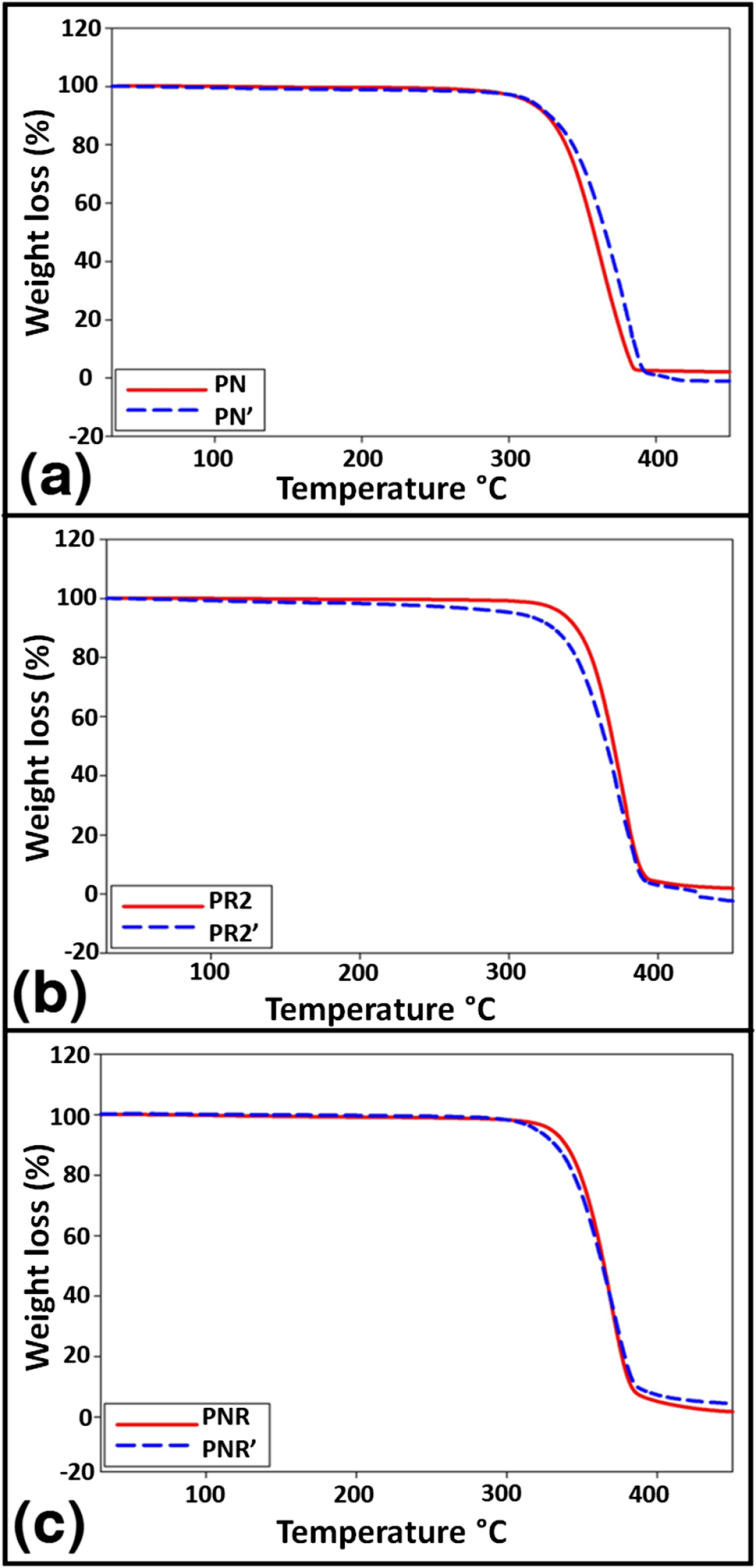
Thermogravimetric analyses of the biodegraded biocomposites; **a** PN, **b** PR, **c** PNR

To further study the effect of biodegradation on the thermal stability of the biocomposites, three biodegraded samples were tested (tagged as PN’ (biodegraded PN), PR2’ (biodegraded PR2) and PNR’ (biodegraded PR2)). Figure 4 presents the thermal behaviour of the biodegraded samples for temperatures between 30 and 450 °C. The PN’ showed a higher thermal stability than the PN which is linked to the stability of cellulose. On the other hand, the PR showed a higher thermal stability than the PR’ which is associated with the biodegradation and the poor thermal stability of NR. At 360 °C, the PN, PN’, PR2 and PR2’ lost about 40, 55, 28 and 40% of their total mass, respectively. The PNR and PNR’ demonstrated very similar thermal behaviour, indicating the thermal stability of the PNR despite the structural changes brought about by the biodegradation process. This is especially suitable in biodegradation applications deployed in tropical and/or hot countries, e.g. Malaysia.

### Morphological analyses

**Fig. 5:**
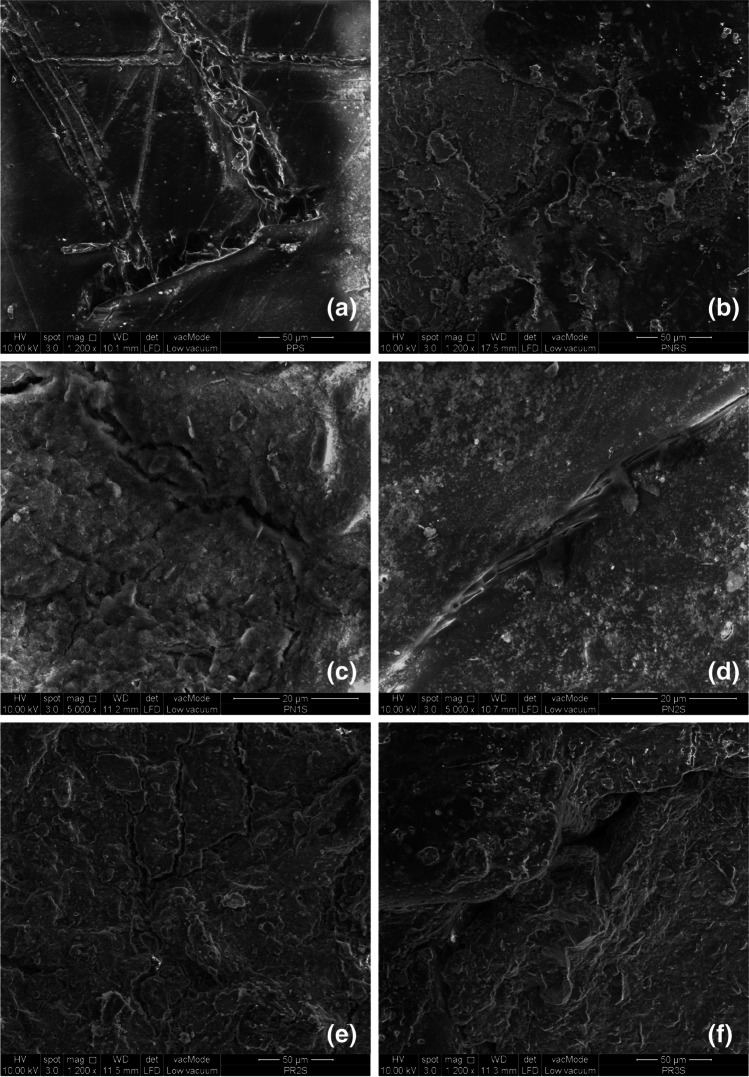
Scanning electron micrographs after 2160 h of soil burial; **a** PLA, **b** PNR, **c** PN1, **d** PN2, **e** PR2, **f** PR3

The effect of biodegradation on the surface of the biocomposites after 2160 h of burial in soil was analysed using SEM micrographs (Fig. 4). Partial biodegradation of the PLA granules was observed which was due to microbial action rather than physical discharge (Shogren et al. 2003). The presence of the cracks and holes confirmed the biodegradation process (Brzeska et al. 2022). A greater number of cracks was observed on the biocomposites with higher rates of biodegradation (i.e. PLA, PN3 and PR3). In addition to the cracks, the shrinkage of the biocomposites also facilitates the exposure of the CNP to moisture and its surrounding area (Harmaen et al. 2015). Due to the reinforcement, compared to the PLA, fewer number of cracks were observed on the PNR surface resulting in a reduction in the biodegradation rate. The PNR is an appropriate fertiliser encapsulating material due to its suitable biodegradation process. Moderate biodegradation of the PNR can also stabilise and control the release of the fertiliser nutrients in soil (Fig. 5).

### Rate of biodegradation and water absorption

As a fertiliser encapsulating material, it is critical to determine the time required for complete the biodegradation of the PNR and the corresponding water absorption rate. The soil burial and the water absorption test results were best fitted to a cubic polynomial model (Eq. 3), where *Y* is $\frac{weight\;loss\%}{water\;absorption\%}$; *X* is the time factor; *Y*_0_, *a*, *b* and *c* are the four coefficients used in the cubic model. The applicability of the polynomial model was examined by assessing the regression coefficient (*R*^2^). The results are presented in Table 2.

**Table 2:** Results of the cubic polynomial regression model for the soil burial test

Test type	Biocomposite	*Y*_0_	*a*	*b*	*c*	*R*^2^
Soil	PNR	0.150	− 0.0045	0.0000052	− 0.0000000013	0.85
	PLA	− 0.028	− 0.0019	0.0000032	− 0.00000000078	0.92
Water	PNR	1.190	0.0096	− 0.0000083	0.0000000021	0.81
	PLA	0.089	0.00028	0.00000099	− 0.00000000041	0.79

3 $$Y={Y}_{0}+aX+{bX}^{2}+{cX}^{3}$$

It is found that almost 3062 and 3863 h of soil burial is required for complete (i.e. 100%) biodegradation of the PNR and PLA, respectively. The biodegradation process is considered *fast* as compared to similar studies reported in the literature (Jin et al. 2022, 37, Sharma et al. 2021). This was directly linked to the test conditions (i.e. high humidity). The biodegradation rates of the PNR and PLA were measured to be about 0.15% and 0.03% per hour, respectively. In other words, the PNR, as an encapsulating material, can last for nearly 3072 h under tropical conditions, boasting a moderate biodegradation rate of about 0.15%/h. The water absorption rate was measured to be 1.2%/h and 0.08%/h for the PNR and PLA, respectively. Slightly higher biodegradation and water absorption rates are linked to the presence of the amorphous regions (Kovács and Tabi 2011, Dogu and Kaynak 2016, Zamir et al. 2022). It was understood that the CNP accelerates while the NR moderates the biodegradation of the PLA biocomposites. This is a further confirmation of the optimum composition used in the PNR biocomposites.

## Conclusions

In this study, indoor biodegradation, thermal degradation and water absorption processes for PNR biocomposites were investigated. The results were best described by a cubic polynomial model. The PNR biocomposites were observed to be biodegradable under natural conditions. Cracks and shrinkage in the biocomposites’ surfaces accelerated the exposure of the CNP to moisture and its environment. Amorphous regions in CNP were realised to play a key role in accelerating the PNR biodegradation. An increase in the biodegradation rate was also linked to the test conditions (i.e. high humidity). The CNP accelerated while the NR moderated the biodegradation rate of the PNR biocomposites. The PNR demonstrated an acceptable water resistance when prepared with the optimum compositions. A composition comprising 3 wt% CNP and 15 wt% NR was observed to be the optimum amount of reinforcement in order to minimise the PLA’s water adsorption. An excess amount of NR in the PR4 biocomposites affected its morphology and led to a higher water absorption capacity. An improvement in the PLA thermal stability was achieved following the integration of both the CNP and NR. Labour and application costs may be reduced by using PNR as a fertiliser encapsulating material by eliminating the need for multiple fertiliser applications. Prolonged nutrient release may provide more uniform plant nutrition, better growth and improved plant performance. Master-batch preparation of the PNR/fertiliser is therefore of great importance in future studies and investigations. The future studies include analysing the interaction between PNR/fertiliser master-batch and soil.

## Data Availability

All data, raw and analysed, have been stored in the university repository and are available upon request. They are not made public due to contractual agreement.
